# A Case of Advanced Keratoconus Treated Effectively With Double Corneal Allogeneic Ring Segments

**DOI:** 10.7759/cureus.99458

**Published:** 2025-12-17

**Authors:** Yusuke Hara, Takahiko Hayashi, Satoru Yamagami

**Affiliations:** 1 Ophthalmology, Nihon University School of Medicine, Itabashi-ku, JPN

**Keywords:** cairs, corneal allogeneic intrastromal ring segments, corneal transplantation, ctak, keratoconus

## Abstract

In this case report, we aim to describe the use of double-ring segments to treat a case of keratoconus in corneal allogenic intrastromal ring segments (CAIRS). A 23-year-old male with advanced keratoconus presented with decreased vision and agreed to be treated by CAIRS surgery. A modified CAIRS was performed with two ring segments inserted into the upper and inferior tunnels. A significant corneal flattening was observed during an early postoperative period, resulting in an improvement of visual acuity without any complications for more than six months following the procedure. In this case, CAIRS, with the use of double segments in the tunnel, was effective in terms of the ability to flatten the corneal curvature and result in a dramatic improvement in visual acuity.

## Introduction

Recently, minimally invasive procedures such as Bowman’s layer transplantation [1] and corneal allogenic intrastromal ring segments (CAIRS) [2] have been developed and are now widespread worldwide. CAIRS is a type of corneal transplantation involving the insertion of allogeneic, ring-shaped donor corneal tissue. It involves implantation of allogeneic corneal tissue into the periphery of the keratoconic cornea, resulting in flattening of the cone. In most keratoconus cases with asymmetrical steepening, only one segment of the CAIRS graft has been used within 160 degrees. In this manuscript, we would like to investigate a favorable outcome of an advanced keratoconus patient treated with CAIRS using double-ring segments. This report aims to provide guidance for surgeons in determining how to plan their own CAIRS procedures. In advanced ectasia with extremely high K values, a single segment may provide insufficient biomechanical effect; therefore, this report highlights the use of dual-segment CAIRS as a strategy to enhance corneal flattening.

## Case presentation

A 23-year-old male college student had noticed progressive vision loss for approximately one year and visited a local clinic. He was then referred to our department for further evaluation and treatment. The best-corrected visual acuity was 0.1 (0.7×S−10.0D: C−2.5D × 95°) in the right eye and 0.03 (n.c.) in the left eye. Ectasia screening with anterior segment optical coherence tomography (OCT) confirmed keratoconus in both eyes. Steep keratometry values (Ks) were 54.7D in the right eye and 69.7D in the left eye. Both eyes were diagnosed as keratoconus, with the right eye at stage 3 and the left eye at stage 4 according to the Amsler-Krumeich classification. Pachymetry showed the thinnest corneal thickness of 376 µm, consistent with advanced ectasia. Preoperative corneal cross-linking had not been performed because, in our institute, corneal collagen cross-linking (CXL) is typically considered only after achieving maximal refractive improvement through CAIRS. The patient reported progressive blurred vision affecting daily activities and had previously tried hard contact lenses (HCL) but discontinued them due to lens-related discomfort. Because the keratoconus was already advanced at the initial presentation, the patient wished to avoid invasive lamellar or penetrating keratoplasty. Therefore, CAIRS was selected as a tissue-preserving alternative expected to provide meaningful visual improvement. Two months after the initial visit, CAIRS surgery was scheduled to treat the more severely affected left eye. 

Surgical technique

**Figure 1:**
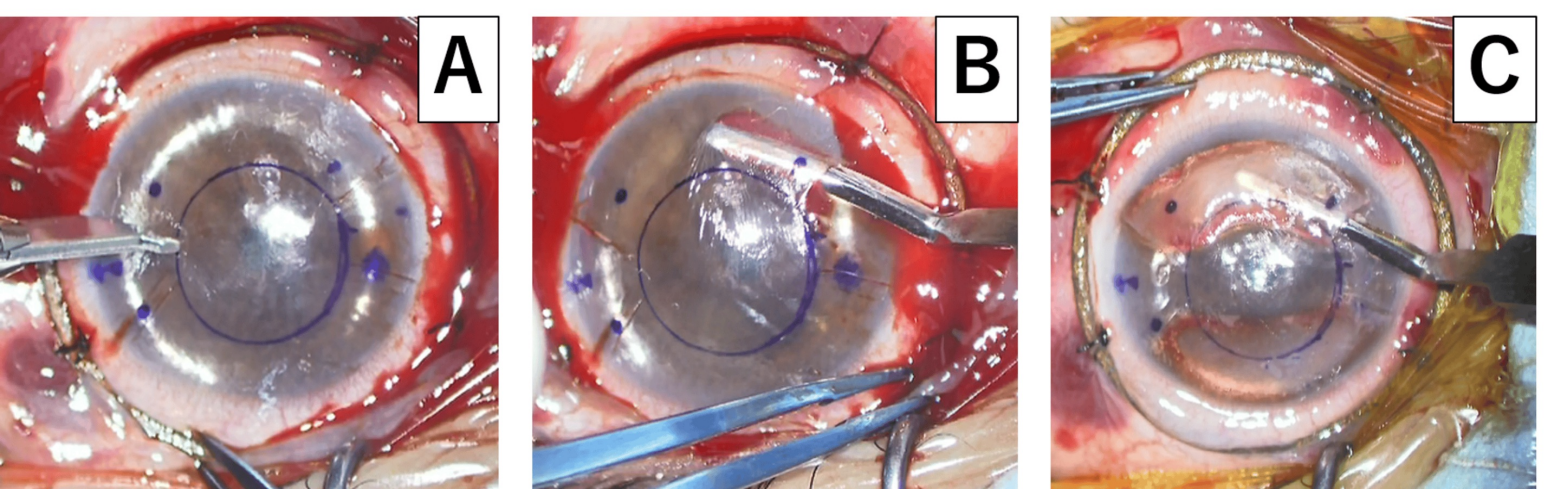
Intraoperative image during manual CAIRS surgery A: After air was injected into the anterior chamber to stabilize intraocular pressure, a tunnel entry was created with a calibrated knife set to a depth of approximately 250 μm. B: The stromal tunnel was then manually dissected across the cornea using a spatula. C: Two allogenic CAIRS grafts were gently advanced into the pocket from the incision site with the same spatula. AC: Anterior chamber; CAIRS: Corneal allogenic intrastromal ring segments

The donor corneal tissue (Optigraft; Lions World Vision Institute, Tampa, FL, USA) was trephined with a double-blade system (Jacob CAIRS trephine, Madhu Instruments, New Delhi, IND) [3]. Maintaining adequate ocular rigidity was essential for accurate tunnel formation; therefore, a Flieringa ring was sutured with four 7-0 silk threads, and the anterior chamber was filled with air. Following the technique described in our previous paper [4], the incision was initiated at approximately 250 μm using a calibrated guarded blade, and a stromal channel (inner diameter = 5 mm; width ≈ 3 mm) was manually dissected with a customized CAIRS spatula (Inami, Tokyo, JPN) (Figures 1A-1B). Both segments were intended to be positioned within the same stromal plane at approximately 250 μm. Although minor variation is inherent in manual dissection, intraoperative OCT confirmed that the two grafts remained within the same lamellar plane without vertical separation. The mid-peripheral corneal thickness should be at least 500 μm to allow creation of a 250-μm stromal incision. The CAIRS graft was inserted into the tunnel using the CAIRS spatula (Figure 1C). The key point in this case was that two grafts were inserted, both superiorly and inferiorly, due to the severity of the disease. The decision to place two segments was based on the marked degree of steepening, where a single segment was unlikely to provide sufficient flattening.

After implantation, the location of the graft was adjusted using the reverse Sinsky hook. Intraoperative OCT (Artevo 800; Carl Zeiss Meditec, Jena, DEU) was used to confirm the depth of the incision and implanted tissue. Finally, the tunnel was closed by 10-0 nylon (Mani Inc., Tochigi, JPN). These stitches were removed within two weeks after the surgery. A subconjunctival injection of 0.4 mg of betamethasone (Rinderon) was administered. 

Postoperative examination

**Figure 2:**
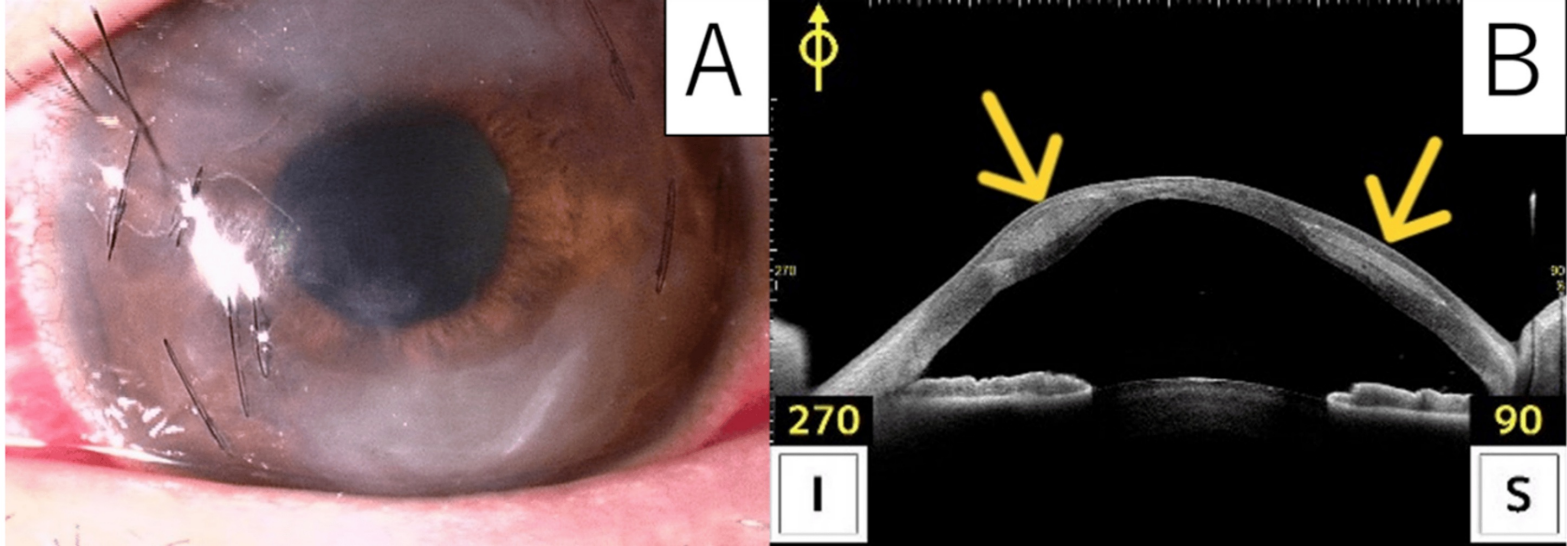
Images after implantation of the double segment CAIRS grafts A: Slit lamp photograph of the day after CAIRS surgery shows one segment in the superior and the other segment in the inferior. Stromal edema was shown around the two tunnels, including both segments. B: One week after CAIRS, anterior segment OCT showed that the ring segments were inserted in the proper position without extrusion. CAIRS: Corneal allogenic intrastromal ring segments; OCT: Optical coherence tomography

**Figure 3:**
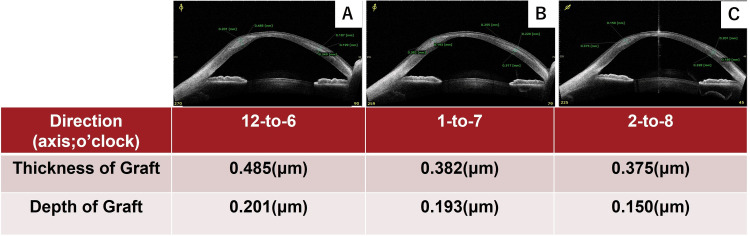
Results of graft thickness and depth in three directions one month after CAIRS Anterior segment OCT confirmed stromal implantation depths of 201 μm at 6 o’clock (A), 193 μm at 7 o’clock (B), and 150 μm at 8 o’clock (C). The graft thickness at each respective location is illustrated in the figure.

The day after surgery, there was a Descemet fold and stromal edema around the graft, and the corneal surface was rough, but there was no erosion (Figure 2A). One week after CAIRS, anterior segment OCT showed that the ring segments were inserted in the proper position without extrusion (Figure 2B). 'Proper position' is defined as the complete intrastromal placement with continuous stromal coverage on OCT, without epithelial elevation or gaping at the incision. 'No extrusion' is defined as the presence of at least 80 μm of stromal tissue above the graft and the absence of graft exposure at the entry site. The visual loss and eye pain caused by these conditions improved by the following week’s examination. Graft depth and thickness were still good one month after CAIRS (Figure 3).

Postoperative course

**Figure 4:**
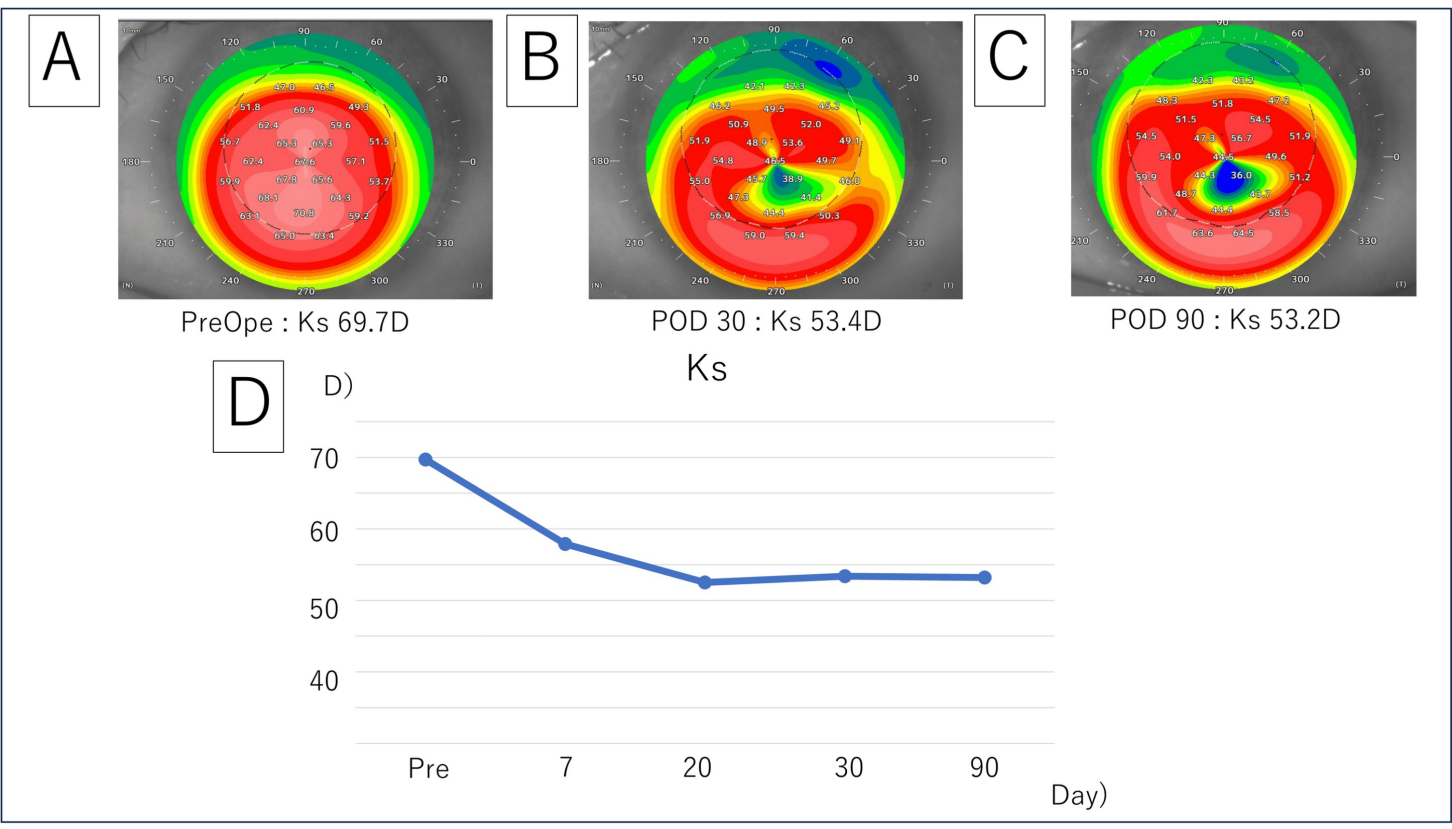
A topographic change after the implantation of the double segment CAIRS grafts A: The preoperative Ks of the left eye show a steep value at about 69.7 D; B: Ks has notably decreased to 53.4 D on day 30; C: The Ks value remained stable on day 90; D: A graph indicating the corneal curvature (Ks) shows that the postoperative corneal curvature significantly improved compared to the preoperative values. D: Diopters; Ks: Steep keratometry; PreOpe: Preoperative; POD: Postoperative day

Postoperative treatment included 1.5% levofloxacin (Cravit), betamethasone (Sanbetason), and 2% rebamipide ophthalmic solution (Mucosta) four times daily for three months and was gradually tapered. The patient was examined routinely five to seven days postoperatively and at intervals of one, three, six, and 12 months. At the first postoperative week visit, visual acuity was 0.5, and Ks was 57.9D. At the first postoperative month visit, visual acuity was 0.7, and Ks was 53.4D. At the third postoperative month, visual acuity was 0.8, and Ks was 53.2 D. The corneal curvature improved postoperatively (Figure 4).

## Discussion

Keratoconus is a progressive ectatic disorder that often begins in adolescence and can lead to significant visual impairment [5]. In contrast, middle-aged patients tend to show less progression than younger individuals. With the development of anterior segment OCT, the prevalence of keratoconus has been reported to be higher than previously expected [6].

Although this is a sight-threatening progressive disease, there have been limitations in available treatment options. Over decades, many advanced keratoconus eyes have been treated by penetrating keratoplasty, or deep anterior lamellar keratoplasty (DALK). Without such special treatments, patients often had to rely on HCL. However, the fitting of HCL can often be uncomfortable [7]. Thus, there have been no moderate treatment options for keratoconus.

Corneal collagen crosslinking is a surgical intervention designed to halt the progression of keratoconus. This technique was first introduced by Wollensak et al. [8]. Since its introduction, CXL has become the only established method capable of biomechanically stabilizing the cornea in keratoconic eyes. Although it effectively prevents further ectatic progression, its impact on visual improvement remains limited.

In 2000, Colin et al. [9] first presented their outcome about the modulation of corneal topography and improved visual acuity in keratoconus using synthetic polymethylmethacrylate (PMMA) intrastromal corneal ring segments (ICRS). But ICRS has synthetic segment-specific complications such as extrusion, intrusion, migration, infection, and acute stromal necrosis [10].

Recently, the CAIRS was developed by Dr. Soosan Jacob [2]. Although the results of this procedure, which had already been reported, look excellent, this procedure normally requires the use of a femtosecond laser, which is expensive. Thus, we came to develop a manual technique without the use of a femtosecond laser and reported significant improvements in best corrected visual acuity (BCVA), maximal keratometry (Kmax), and corneal higher-order aberrations, although the sample size was small (n=5) [11]. Although femtosecond-assisted CAIRS provides a highly reproducible tunnel geometry, its cost limits availability in many regions. Manual CAIRS offers a low-cost alternative, and in this case, intraoperative OCT ensured accurate tunnel depth and graft position despite the inherent variability of manual dissection.

There are two main points that we would like to emphasize in this article. One of the strengths of our article was the advantage of avoiding DALK, although the patient showed a strong topographic steep change. Some surgeons might decide to perform DALK in such an advanced case. The DALK might have several complications, such as intraoperative perforation of Descemet’s membrane with conversion to penetrating keratoplasty (PKP), double-chamber formation, and suture-related complications.

The second strength of our article is to prove the effectiveness of our manual CAIRS technique without the use of expensive instruments such as the femtosecond laser. The degree of flattening observed in this case appears greater than that typically reported with standard single-segment CAIRS, including a previously published manual technique [12]. The biomechanical effect of dual-segment CAIRS can be understood as a symmetric redistribution of stromal volume, enhancing central flattening compared with a single segment. This can be explained by the Barraquer thickness law. Specifically, thinning the central area flattens it, providing a myopia-correcting effect, while thinning the peripheral area steepens it, providing a hyperopia-correcting effect. In CAIRS surgery, inserting a graft into the peripheral cornea produces a flattening effect at its center, and this effect is proportional to the volume of the transplanted graft [13]. Bteich et al. reported that the more severe the keratoconus, the thicker the segment and the more superficial the implantation [14]. In fact, we were able to obtain the flattening effect over 10D by the use of two-segment implantations of grafts. And even when two grafts are inserted into a channel, as in this case, if the depth and width are properly created, the effect of CAIRS is achieved without complications such as extraction or migration, so far. This case demonstrates that even in eyes with severe steepening, dual-segment CAIRS may offer a meaningful, tissue-preserving alternative to DALK when appropriately planned.

Limitations

This report describes a single case study with detailed postoperative data available only up to three months. Although follow-up examinations were performed for 12 months, only short-term outcomes are presented here. To confirm long-term stability and reproducibility, extended follow-up over 12 to 24 months or longer and evaluation across multiple cases are necessary. Biomechanical parameters, visual quality measures, and patient-reported outcomes were not assessed, which limits the ability to fully characterize functional improvement. Future research should aim to collect objective parameters such as endothelial cell counts, pachymetry, higher-order aberrations, corneal morphology, and patient-reported outcomes, as well as include comparative studies with other treatment approaches, i.e., CXL plus ICRS, DALK, and femto-CAIRS.

## Conclusions

Although this report describes a single case, the substantial corneal flattening achieved with a dual-segment CAIRS configuration suggests that customized CAIRS designs may be beneficial in selected cases of advanced keratoconus. The use of two allogeneic ring segments allowed effective biomechanical modulation while preserving native corneal tissue and avoiding more invasive keratoplasty. This approach may expand the treatment options for patients with severe ectasia who wish to defer or avoid deep anterior lamellar keratoplasty or penetrating keratoplasty. Larger studies with longer follow-up are needed to evaluate long-term stability, safety, and optimal indications for dual-segment CAIRS.

## References

[1] van Dijk K, Liarakos VS, Parker J, Ham L, Lie JT, Groeneveld-van Beek EA, Melles GR (2015). Bowman layer transplantation to reduce and stabilize progressive, advanced keratoconus. Ophthalmology.

[2] Jacob S, Patel SR, Agarwal A, Ramalingam A, Saijimol AI, Raj JM (2018). Corneal allogenic intrastromal ring segments (CAIRS) combined with corneal cross-linking for keratoconus. J Refract Surg.

[3] Jacob S, Agarwal A, Awwad ST, Mazzotta C, Parashar P, Jambulingam S (2023). Customized corneal allogenic intrastromal ring segments (CAIRS) for keratoconus with decentered asymmetric cone. Indian J Ophthalmol.

[4] Hayashi T, Hara Y, Sunouchi C (2025). A manual technique for corneal allogeneic intrastromal ring segments without a femtosecond laser. Cornea.

[5] Fahd DC, Alameddine RM, Nasser M, Awwad ST (2015). Refractive and topographic effects of single-segment intrastromal corneal ring segments in eyes with moderate to severe keratoconus and inferior cones. J Cataract Refract Surg.

[6] Zadnik K, Barr JT, Edrington TB (1998). Baseline findings in the Collaborative Longitudinal Evaluation of Keratoconus (CLEK) Study. Invest Ophthalmol Vis Sci.

[7] Papaliʼi-Curtin AT, Cox R, Ma T, Woods L, Covello A, Hall RC (2019). Keratoconus prevalence among high school students in New Zealand. Cornea.

[8] MacIntyre R, Chow SP, Chan E, Poon A (2014). Long-term outcomes of deep anterior lamellar keratoplasty versus penetrating keratoplasty in Australian keratoconus patients. Cornea.

[9] Wollensak G, Spoerl E, Seiler T (2003). Riboflavin/ultraviolet-a-induced collagen crosslinking for the treat ment of keratoconus. Am J Ophthalmol.

[10] Colin J, Cochener B, Savary G, Malet F (2000). Correcting keratoconus with intracorneal rings. J Cataract Refract Surg.

[11] Kanellopoulos AJ, Pe LH, Perry HD, Donnenfeld ED (2006). Modified intracorneal ring segment implantations (INTACS) for the management of moderate to advanced keratoconus: efficacy and complications. Cornea.

[12] Parker JS, Dockery PW, Parker JS (2021). Flattening the curve: manual method for corneal allogenic intrastromal ring segment implantation. J Cataract Refract Surg.

[13] Barraquer JI (1989). Basis of refractive keratoplasty — 1967. Refract Corneal Surg.

[14] Bteich Y, Assaf JF, Mrad AA, Jacob S, Hafezi F, Awwad ST (2023). Corneal allogenic intrastromal ring segments (CAIRS) for corneal ectasia: a comprehensive segmental tomography evaluation. J Refract Surg.

